# eHealth interventions to support caregivers of people with dementia may be proven effective, but are they implementation-ready?

**DOI:** 10.1016/j.invent.2019.100260

**Published:** 2019-07-24

**Authors:** Hannah L. Christie, Jennifer L. Martin, Jade Connor, Huibert J. Tange, Frans R.J. Verhey, Marjolein E. de Vugt, Martin Orrell

**Affiliations:** a Department of Psychiatry and Neuropsychology and Alzheimer Centre Limburg, School for Mental Health and Neurosciences, Maastricht University, Maastricht, the Netherlands; b NIHR MindTech MedTech Co-operative, The Institute of Mental Health, Nottingham University Innovation Park, Nottingham, United Kingdom; c Department of International Health, CAPHRI School for Public Health and Primary Care, Maastricht University, Maastricht, the Netherlands; d Department of Family Practice, CAPHRI School for Public Health and Primary Care, Maastricht University, Maastricht, the Netherlands; e Institute of Mental Health, University of Nottingham, Nottingham, United Kingdom

**Keywords:** Dementia, Caregiver, Internet, eHealth, Implementation

## Abstract

**Objectives:**

A variety of health services delivered via the Internet, or “eHealth interventions,” to support caregivers of people with dementia have shown evidence of effectiveness, but only a small number are put into practice. This study aimed to investigate whether, how and why their implementation took place.

**Methods:**

This qualitative study followed up on the 12 publications included in Boots et al.'s (2014) widely cited systematic review on eHealth interventions for informal caregivers of people with dementia, in order to explore further implementation into practice. Publicly available online information, implementation readiness (ImpRess checklist scores), and survey responses were assessed.

**Findings:**

Two interventions were freely available online, two were available in a trial context, and one was exclusively available to clinical staff previously involved in the research project. The remaining seven were unavailable. All scores on the ImpRess checklist were at 50% or lower of the total, indicating that the interventions were not ready to implement at the time of the Boots et al. (2014) review, though some interventions were scored as more implementation-ready in subsequent follow-up publications. Responses to the survey were received from six out of twelve authors. Key learnings from the survey included the importance of the involvement of stakeholders at all stages of the process, as well as the flexible adaptation and commercialization of the intervention.

**Conclusions:**

In general, low levels of implementation readiness were reported and often the information necessary to assess implementation readiness was unavailable. The only two freely available interventions had long-term funding from aging foundations. Authors pointed to the involvement of financial gatekeepers in the development process and the creation of a business model early on as important facilitators to implementation. Future research should focus on the factors enabling sustainable implementation.

## Highlights

• Most interventionscouldno longer be found.
• Most interventions reported low implementation readiness.
• Available interventions made use of long-term funding.
• They were not found to have developed self-sustaining business models.
• More research is needed on business models and contextual factors.

## 1 Introduction

Globally about 50 million people are living with dementia. This number is expected to triple by 2050 (Werner et al., 2017). Informal caregivers often experience substantial physical and psychological problems as they care for people with dementia (Brodaty and Donkin, 2009; Ma et al., 2017).

Regarding psychosocial interventions to support informal caregivers of people with dementia, Gitlin et al. (2015) reported that less than 3% of these interventions that are effective in research studies, are put into practice. This is due to a lack of research into facilitating and impeding factors for the continuation of use in clinical practice, insufficient theories to understand implementation challenges, lack of funding, and ill-fitting financial frameworks for sustaining interventions. One type of psychosocial intervention that may help informal caregivers maintain their wellbeing and so cope better for longer, is eHealth. eHealth interventions can be defined as ‘treatments, typically behaviorally based, that are operationalized and transformed for delivery via the Internet’ (Ritterband et al., 2006). These interventions often include self-guided, interactive, and personalized programs. Reported benefits to using eHealth interventions (not specifically for caregivers of people with dementia) over traditional face-to-face interventions include relatively easy scale-up, wide accessibility despite differences in the socioeconomic and demographic backgrounds of users, personalization, instant delivery, and real-time feedback (Kaplan and Stone, 2013).

eHealth has so far generated much enthusiasm from funding and policy institutions. In the Netherlands, the national dementia action plan, the *Deltaplan Dementie* (van Rijn, 2015), includes the promotion of innovations in eHealth as one of its goals to improve dementia care practices for both people with dementia and caregivers. In its *eHealth Action Plan 2012–2020*, the European Commission asserted that eHealth enables a more ‘citizen-centric’ system of care by increasing socioeconomic inclusion, patient empowerment, and access to services and information (European Commission, 2012). The Council of the European Union called for discussions on the use of eHealth and other tools to support and care for people with dementia and their caregivers (Council of the European Union, 2015). Moreover, one of the target goals of the WHO action plan for dementia is to ‘facilitate access to affordable, evidence based resources for carers to improve knowledge and skills, reduce emotional stress and improve coping, self-efficacy, and health by making use of information and communication technologies such as Internet and mobile phone technologies’ (World Health Organization, 2017). Such action plans have created a political impetus for change, resulting in the allocation of resources for the development and evaluation of eHealth interventions.

Numerous systematic reviews report improvements in informal caregivers' wellbeing through eHealth interventions (Boots et al., 2014; Dickinson et al., 2017; Hopwood et al., 2018; Jackson et al., 2016; Lee, 2015; Parra-Vidales et al., 2017; Scott et al., 2016; Tyack and Camic, 2017). Generally, studies find positive gains in caregivers' self-efficacy, competence, and knowledge about dementia, as well as a reduction of depressive symptoms. Multicomponent interventions (interventions with two or more intervention components; Cantera et al. (2015)) are often more effective than interventions focused on one area alone (Olazarán et al., 2010). Some examples include online self-management courses for dementia, via desktop browser; other examples are apps to deliver and facilitate psychological support from both fellow caregivers and health care professionals.

Christie et al. (2018) showed that the bulk of research on eHealth interventions for caregivers of people with dementia has focused on the trial phase, with almost no studies examining their implementation. Decision-makers looking to implement these interventions also find there is a lack of public awareness and confidence in eHealth, limited evidence of the cost-effectiveness of interventions, lack of legal clarity (especially with regard to data protection and reimbursement), and high start-up costs (European Parliament and the Council of the European Union, 2016). Moreover, implementing eHealth interventions for aging populations has specific challenges, including changes in motor, cognitive, and perceptive abilities with age, in combination with the continuing, fast-paced evolution of modern technologies (Preschl et al., 2011). These implementation barriers hinder the use of eHealth interventions across various fields in practice (Vis et al., 2018).

Instead of doing another systematic review on the effectiveness of eHealth interventions for caregivers of people with dementia, the aim of this study was rather to better understand the implementation trajectories of evidence-based eHealth interventions for informal caregivers of people with dementia and how they can be implemented in practice, by the follow-up of the interventions highlighted in a previous systematic review (Boots et al., 2014).

## 2 Methods

### 2.1 Study selection

Due to this study's aim to follow up on a select sample of evidence-based interventions from a high quality and highly-cited systematic review, only studies from a single systematic review were included. Boots et al. (2014) was selected, as it was widely-cited (171 citations reported by Google Scholar as of January 2019) and presented data on 12 studies of ‘internet-based’ interventions for informal caregivers of people with dementia, published between 1995 and 2013. At the time of its publication, eHealth was an established field with many intervention studies published. Earlier reviews (pre-2014) tended to include more studies of interventions containing technology that is no longer in use or that is incompatible with current software and hardware requirements. On the other hand, the authors propose that a more recent review (between 2014 and 2017, the time of this study's design) would not have allowed for sufficient time to implement the interventions, as it is the authors' understanding that the eHealth implementation process tends to take several years post-efficacy trial. The inclusion criteria for the Boots et al. (2014) review were that the study (i) reported the effects of an intervention; (ii) was Internet-based; and (iii) was aimed at informal (nonprofessional) caregivers; of (iv) people with mild cognitive impairment or dementia. Examples of reviewed eHealth interventions include: Online psychoeducation courses with and without coach, web-based support via video conferencing, caregiver-therapist email support, etc. More details on the included studies' efficacy and outcomes can be found in Boots et al. (2014).

### 2.2 Study design

This is an exploratory, qualitative study using data gathered from following up on a sample of studies, using information publicly available on the internet, information on the implementation readiness reported by the publications, and authors' survey responses.

### 2.3 Data collection

First, data was collected on what information about the 12 eHealth interventions could be found by searching the internet. A data extraction form listing the studies' intervention descriptions, funding, number of citations, availability, follow-up information and survey participation (Appendix A). Furthermore, in order to assess the most recent studies investigating the same or a later version of the intervention, a PubMed and Google Scholar search looking for articles including the intervention name, and published after the included article date, was conducted. Additionally, publications from the involved authors were scanned to assess whether any new publications referenced new iterations of the original intervention (perhaps now under a different name).

Second, implementation readiness of both the original articles included in Boots et al. (2014) review and the follow-up articles identified by the PubMed and Google Scholar search was assessed using the ImpRess checklist, a checklist for evaluating readiness for implementation of manualized interventions (Streater et al., 2016). This instrument was chosen as it was developed to assess whether evidence-based interventions are ready to implement following their efficacy trials, prior to identifying an organizational implementation context. The ImpRess checklist consists of 26 questions (Box 1), grouped into ten themes and scored with 0, 1, or 2 points per question, making for a minimum total score of 0 and a maximum total score of 52. A score of 0 signifies that no information was provided, a score of one signifies that the question was partially answered and a score of two signifies a fully answered question. Themes are not weighted and the total score serves as the indication of implementation readiness. The coding was carried out by author HLC. The ImpRess scores were compiled for both the included interventions from Boots et al. (2014), as well as for their follow-up studies identified by the internet search, described above (if applicable). Filling in the ImpRess checklist for these subsequent studies was done to take into account the fact that implementation information is sometimes not reported in efficacy studies. Assessing the follow-up publications was deemed necessary to provide more insight into how these interventions develop and how implementation readiness may or may not have been reported since the effectiveness study. This is because effectiveness studies do not provide much information about implementation issues and subsequent publications often do. It was thought that all checklist themes, including the themes measuring the employee and manager support were a relevant and necessary part of all eHealth interventions, including the psychoeducation platforms. This is at the least true for the reason that the software must be consistently updated, but also for keeping the information provided up-to-date.

Box 1 **ImpRess questions.**

**Table:** 

Theme	Question	
Motivation	1.	Does the existing evidence suggest the intervention is likely to be cost effective?
	2.	Does the existing evidence suggest the intervention is likely to be effective for the primary outcome?
	3.	Does the existing evidence suggest the intervention is likely to be effective for other key outcomes?
	4.	Are there other benefits for the patient (qualitative)?
	5.	Are there benefits for the organization?
Theory of change	6.	Are the outcomes clearly defined?
	7.	Is how the intervention works clearly defined?
	8.	Is the design suitable for the kind of intervention (RCT)?
	9.	Is there a coherent theoretical base?
Implementation	10	Is the intervention standardized?
	11.	Can it be widely implemented into practice (following on from a research setting)?
Experience	12.	Are the skills and experience of the person delivering the intervention clearly described?
	13.	Is there monitoring of the delivery (attendance/adherence) of the intervention?
Planning consultations	14.	Is the amount of time necessary to set up the intervention specified?
	15.	Is the planning and setting up of the sessions clearly defined?
Delivery collaborations	16.	Does it specify the amount of time required for each session and for the duration of the program?
	17.	Are the potential facilitator and barriers to the delivery of the intervention described?
Manager support	18.	Is the level of managerial support described during the intervention/evaluation?
Employee support	19.	Is the level of support required by staff members to deliver the intervention described?
Resources	20.	Are the resources required to deliver the intervention specified?
	21.	Are the training costs specified?
	22.	Are the training materials specified?
	23.	Are there manuals for the intervention?
	24.	Are the materials easy to source?
Population characteristics	25.	Are the population characteristics specified?
	26.	Does it specify who benefits most from the intervention?

Alt-text: Box 1

Finally, a survey of open-ended questions about researchers' experiences with the development and implementation of their eHealth interventions was developed, examining the current status of the intervention, the latest evidence of the effectiveness, and perceived facilitators and barriers to its development and implementation. The survey was piloted and reviewed by a researcher from Maastricht University involved in the development of an eHealth intervention for informal caregivers of people with dementia, not included in the review. Based on the feedback from this piloting, necessary modifications to the survey (including more specifically worded questions) were made. Box 2 contains the questions included in the final version of the survey. The survey was sent to the authors via email, and respondents were given two weeks to complete the survey. Multiple reminders were sent after the two-week period to encourage response. If there was no response by the first author, the last author was then invited to complete the survey and reminders were sent after two weeks. Researchers who did not agree to the informed consent were not included in this study.

Box 2 **Survey questions.**

1. Is the intervention currently available to purchase or otherwise obtain?
2. If yes, how can someone get access the intervention? Who is this intervention available to?
3. Are you aware of it being used in practice now (please provide details)?
4. Is there any additional evidence (besides the effectiveness paper included in the review by Boots et al. (2014)) on the intervention's effectiveness?
5. Barriers to the development of the intervention
6. Barriers to the implementation of the intervention
7. Facilitators to the development of the intervention
8. Facilitators to the implementation of the intervention
9. How widespread is its use?
10. Which countries is it used in?
11. Recommendations for developing and implementing E-health interventions for caregivers of people with dementia (lessons learned)
12. Did you use any theoretical models for the development/implementation of your intervention (for example, the MRC framework)? If so, what were your experiences with the model(s)?

Alt-text: Box 2

### 2.4 Data analysis

By compiling information on the interventions' content, funding, number of citations, availability, follow-up information and survey participation (Appendix A), the authors compared these characteristics in the interventions that could still be found online, versus these characteristics in the interventions that could not be found. When comparing the two groups regarding these characteristics, the authors attempted to discern whether there were any characteristics that typified either group, and whether they might contribute to enduring intervention use. The survey responses were compiled and analyzed using thematic content analysis (Evers, 2015; Thomas, 2006). Two researchers (JC and HC) performed the analysis independently, using inductive reasoning and constant comparison, in order to identify categories across the questions asked in the survey. In doing so, open codes were applied to survey responses. After thorough reading, categories and higher-order themes were constructed by merging the open codes. A consensus meeting was held with the two analyzers and MdV to discuss and resolve any discrepancies in the two analyses. Analysis was performed with the software package Atlas.ti (Version 1.0.14 for Apple Macintosh) and mind maps were created.

## 3 Results

### 3.1 Available online information

The initial internet search investigated the current status and focus of the interventions listed in the Boots et al. (2014) review (see Appendix A). If no up-to-date information could be found through a Google search using the intervention's and/or author's name, it was assumed that the intervention was no longer available. If up-to-date information could be found, the interventions were referred to as ‘still-available’. This search showed that websites of five of the interventions appeared to be up and running: four under the same intervention name (Coulehan, 2011; Ducharme et al., 2011; Glueckauf et al., 2004; Lewis et al., 2010) and one under a changed name (Marziali and Garcia, 2011), while no up-to-date information could be found for the remaining seven interventions. The content of these still–available interventions could be grouped into web- and peer-based support (1) and psychoeducation (4). The interventions' associated publications had been cited a median of 46 times, ranging between 5 and 322 (as of November 2018). Funding for the interventions (and their associated publications) could be categorized into six groups: National health and aging institutes (4), regional aging institutes (3), university department grants (2), national ministry of economic affairs (1), Alzheimer's foundation (1), and unknown (2).

The PubMed search for subsequent publications using the intervention's name resulted in a total of five follow-up publications (Appendix A). Two papers were about the randomized controlled trials (RCTs) of adapted versions of the interventions (Griffiths et al., 2015; Van Mierlo et al., 2015), one paper was a qualitative analysis based on the same study as the original included publication (Chiu and Eysenbach, 2011), one publication was a study examining clinicians' and clients' satisfaction with intervention training and delivery of a later version of the intervention (Nalder et al., 2018), and one publication was a cost justification analysis (Payton et al., 1995). Otherwise no follow-up publications examining subsequent implementation were found. Of the five interventions with a follow-up paper, two are still available to use (Lewis et al., 2010; Marziali and Garcia, 2011).

### 3.2 Implementation readiness

In order to assess whether the included studies were indeed ready to be implemented at the time of their publication, the studies included in the Boots et al. (2014) review were scored using the Implementation Readiness (ImpRess) checklist (Streater et al., 2016). The ImpRess checklist was derived from a set of criteria for evaluating the quality of reporting of the implementation of workplace interventions, and was adapted using the Medical Research Council framework to assess more implementation barriers for cognitive stimulation therapy (CST). The checklist is a new tool, with little usage and reliability data available, though it demonstrated a 99,4% inter-rater reliability during development (Streater et al., 2016). It was chosen for its unique ability to assess implementation readiness of evidence-based interventions, without requiring the intervention to have yet been implemented in an organizational context, as this is often not yet the case in effectiveness trials. One publication (Coulehan, 2011) could not be accessed - both author Boots and author Coulehan corresponded that they were no longer in possession of the original publication, a conference presentation. The average checklist score was 19. The scores ranged between 13 and 26 (out of a maximum of 52). Overall, the results showed that the publications included in Boots et al. (2014) achieved the highest ImpRess scores for the themes Theory of change, Implementation, and Population characteristics. These publications achieved the lowest ImpRess scores for the themes Manager Support, Employee Support, and Resources (Table 1).

**Table 1:** ImpRess scores.

Author(s)/year from Boots et al., 2014	Total ImpRess score (max score 52)	Summary scores									
		Motivation (max score 10)	Theory of change (max score 8)	Implementation (max score 4)	Experience (max score 4)	Planning consultations (max score 4)	Delivery collaborations (max score 4)	Manager support (max score 2)	Employee support (max score 2)	Resources (max score 10)	Population characteristics (max score 4)
Beauchamp et al., 2005	21	6	8	2	0	0	2	0	0	0	2
Brennan et al., 1995	25	4	8	3	1	1	3	0	1	0	4
Chiu et al., 2009	23	3	8	3	3	0	2	0	0	0	4
Ducharme et al., 2011	21	5	8	3	3	0	2	0	0	0	4
Glueckauf et al., 2004	20	2	8	3	2	1	2	0	0	0	2
Kelly, 2003	8	2	2	2	0	1	0	0	0	0	1
Lai et al., 2013	13	1	7	2	0	2	0	0	0	0	1
Lewis et al., 2010	21	6	8	3	0	0	0	0	0	0	4
Marziali and Garcia, 2011	26	4	8	2	2	2	4	0	0	2	2
Torp et al., 2008	17	4	5	3	0	0	3	0	0	0	2
van der Roest et al., 2010	13	4	5	2	0	0	0	0	0	0	2
Average total score	18,9	3,7	6,8	2,5	1,0	0,6	1,6	0,0	0,1	0,2	2,5
Average percentage max score	36%	37%	85%	64%	25%	16%	41%	0%	5%	2%	64%

The five follow-up studies found through the PubMed and Google Scholar search achieved an average ImpRess total score of 27 (range 8–26), with an average improvement of 5 points with the original article's score (Table 2).

**Table 2:** ImpRess follow-up scores.

Author(s)/year from Boots et al., 2014	Author(s)/year of follow-up article	Total follow-up ImpRess score (max score 52)	Summary scores									
			Motivation (max score 10)	Theory of change (max score 8)	Implementation (max score 4)	Experience (max score 4)	Planning consultations (max score 4)	Delivery collaborations (max score 4)	Manager support (max score 2)	Employee support (max score 2)	Resources (max score 10)	Population characteristics (max score 4)
Brennan et al., 1995	Payton et al., 1995	36	6	8	3	3	1	3	3	1	4	4
Chiu et al., 2009	Chiu and Eysenbach, 2011	24	3	8	3	3	0	3	0	0	0	4
Lewis et al., 2010	Griffiths et al., 2015	26	6	8	3	2	1	2	0	0	0	4
Marziali and Garcia, 2011	Nalder et al., 2018	29	5	8	4	2	2	4	0	0	2	2
van der Roest et al., 2010	Van Mierlo et al., 2015	19	5	6	2	2	0	2	0	0	0	2
Average total		26,8	5,0	7,6	3,0	2,4	0,8	2,8	0,6	0,2	1,2	3,2
Average percentage max score		52%	50%	95%	75%	60%	20%	70%	15%	10%	12%	80%

### 3.3 Survey responses

The overall participation rate for the survey was 6/12 (50%). Five authors filled in the survey, one responded to the email and provided a written update on the intervention in question. Two additional authors declined to participate. No response was received from the remaining four authors, who could not be traced.

The responses to the survey came from authors involved in the interventions tested in Coulehan (2011), Lewis et al. (2010), Marziali and Garcia (2011), Ducharme et al. (2011), and van der Roest et al. (2010), with additional email correspondence from the first author of the Beauchamp et al. (2005) publication. Three of the respondents reported that their interventions (Beauchamp et al., 2005; Lewis et al., 2010; van der Roest et al., 2010) were no longer available for use. Three of the respondents reported that their interventions (Coulehan, 2011; Ducharme et al., 2011; Marziali and Garcia, 2011) were currently still available. Of these interventions that were still available, one (Marziali and Garcia, 2011) had been integrated into a larger portal that develops patient-owned electronic health records, one (Coulehan, 2011) is aided by funding from the National Institute on Aging of the interventions, and one is still being tested in an academic trial setting (Ducharme et al., 2011).

Based on the survey responses, three themes emerged from the researchers' responses to the questions in Box 1. These themes were the ‘Iterative Development Process,’ the ‘Flexible and Personalized Content,’ and the ‘Integrated Delivery of the Intervention’ (Table 3). The themes are illustrated by quotations in the next sections.

**Table 3:** Summary of survey themes and subthemes.

1. Iterative development process	2. Flexible, personalized content	3. Integrated delivery of intervention
a. Early involvement of stakeholders b. Small sample tests c. Non-linearity	a. Tailoring to needs caregiver b. Theoretical models *Standardization* *Social Cognitive Theory* *Stress Process Model* *Cognitive Behavioral*	a. Cooperation from healthcare organizations b. Commercialization and early development of business model c. Funding

#### 3.3.1 Iterative development process

The first theme that arose out of the data is the ‘Iterative Development Process’. When questioned about their experienced implementation facilitators, respondents emphasized the following facilitators: the involvement of stakeholders at all stages of the process: development, evaluation, and implementation.

“*Involve your target audience as much as possible from the earliest stage of development (needs assessment stage)*.”

Respondent A

“*Conduct a pilot-study with few caregivers before a larger study. From the beginning of the project, involve a community organization supporting caregivers to ensure the sustainability of the intervention*.”

Respondent B

When asked about their recommendations for future developers of eHealth interventions for caregivers of people with dementia, respondents recommended a non-linear process for solving technical problems. Some respondents noted that during the research phase, researchers should start with feasibility tests of small samples.

“*Testing user-friendliness and usefulness in one study is not the best thing to do. If people cannot work with the tool, the usefulness will not be salient, because the tool will not be used. Better to test in two separate studies. And test effectiveness only when a product is in the final stage*.”

Respondent C

#### 3.3.2 Flexible and personalized content

The theme of ‘Flexible and Personalized Content’ highlights the importance of the flexible adaptation of the intervention. One recommendation was:

“*Adapt the content and the technology to the target audience as much as possible*.”

Respondent A

Researchers also provided responses to questions inquiring as to the various psychosocial theories that guided content development. One respondent stated that the standardized education model of the intervention in question was no longer used, as new interventions instead emphasized flexibility. In its further development and current iteration, the focus was placed on tailoring intervention content and technology to the caregivers' needs.

“*Since the 2014 publication intervention has been further developed with emphasis on caregivers/patients taking the lead in customizing change behaviors based on their self-defined needs*.”

Respondent D

Other theories that were reported to have influenced the intervention's design and content were Social Cognitive Theory, Stress Process Model and Cognitive Behavioral Psychology.

#### 3.3.3 Integrated delivery of the intervention

The last theme from the survey responses regards those facilitators mentioned by the respondents that related to the ‘Integrated Delivery of the Intervention’. One recurring facilitator was the continuing involvement of all stakeholders in ensuring the interventions be sustainably implemented into practice and reach the informal caregivers, through collaboration with health organizations, research institutions, non-governmental organizations, or private companies. Survey respondents viewed this integrated delivery of their interventions as important and considered the fragmentation of care systems to be a barrier to doing so. Commercialization and having a business plan were identified as facilitators to implementation.

“*A business model that promotes shared responsibility for maintenance of [intervention C], instead of responsibility with one party*.”

Respondent C

“*We believe that only by commercialization will it be possible to disseminate and mobilize use of [intervention D]*.”

Respondent D

“*There were several thoughts about ways to get it out to the public, but I don't believe they materialized. I no longer work for the company that created it, and in fact, the company has folded*.”

Respondent E

“*Fragmentation and changes within care landscape, makes it difficult to get an up to date overview of available services. Information on care organizations necessarily to develop demand driven ontology and algorithms are not always easy available. Collaborations with researchers, end-users and developers/programmers is not easy because of different perspectives*.”

Respondent C

One respondent mentioned organizational sponsorship as an important facilitator, but also said that the professionals' lack of training on eHealth interventions or reluctance to depart from traditional interventions could be a barrier to collaboration. The unfamiliarity of both caregivers and staff with the intervention technology was seen as a barrier to implementation.

“*The greatest barrier has been healthcare professionals and organizations reluctance to use technology (largely group video conferencing) to deliver online evidence-based intervention programs – preferring to continue to use interventions aligned with their professional training*.”

Respondent D

“*Based on my experience with the [intervention F] trial, and thinking beyond the trial to broader dissemination/implementation, I think some kind of organizational sponsorship – a healthcare company, an insurer, the Alzheimer's Association – is imperative. These kinds of e-education programs make the most sense when they are linked to and integrated with the larger service delivery system*.”

Respondent F

### 3.4 Integrated results

Comparing the results of the internet search and the ImpRess scores, it appeared that, while the intervention with the highest ImpRess score was still available (Marziali and Garcia, 2011), the two next-highest were not (Brennan et al., 1995; Chiu et al., 2009). Of the four studies scoring in the 20–21 range, three were still available and one was not. None of the four publications scoring lower than 20/52 were still available.

Regarding the results of the internet search and the survey responses, the findings indicated that, 3/5 of the authors of the still-available interventions took part in the survey, versus 3/7 of the authors of the discontinued interventions. Based on the available online information and the responses received from the authors, it could be deduced that, of the five still-operating interventions, two were exclusively available through participating in a trial (Ducharme et al., 2011; Lewis et al., 2010), one was only available to select clinical staff previously associated with the research project (Marziali and Garcia, 2011), and two were freely available online (Coulehan, 2011; Glueckauf et al., 2004). The most commonly reported facilitator (mentioned in four out of six responses), developing a commercialization and/or business plan, did not appear to be a guarantee of success. It was mentioned by one of the three respondents with still-available interventions, but also by three out of three of the respondents with interventions that were no longer in use.

Concerning the results of the ImpRess scores and the survey responses, the themes with the lowest average scores on the ImpRess checklist (Manager Support, Employee Support, and Resources) were reflected by survey respondents' answers grouped into the theme ‘Integrated delivery of the intervention’. Here, organizational sponsorship was highlighted as an important facilitator. Respondents also mentioned professionals' lack of training on eHealth interventions as a barrier.

## 4 Discussion

The findings of this study emphasize the key difficulties in translating useful interventions into practice. Only two out of the twelve interventions appeared to be still available to caregivers outside of a trial context, and they were both freely accessible dementia care websites. Three interventions were only available via an ongoing research project. The remaining seven interventions were unavailable or no information was found on their availability. Though it was assumed that the intervention was no longer available, it is possible that the search missed still-available interventions and the assumption of unavailability may well be false. However, it is probable that someone wishing to access the promising, evidence-based intervention based on the information in its efficacy trial study would most likely not be successful. Although this was not mentioned by our survey respondents, it also important to take into consideration that factors unrelated to implementation issues might be influencing the long-term success of some of the interventions. For instance, despite the overall positive effects discovered in the review of Boots et al. (2014), the specific challenges associated with eHealth for elderly populations (including changes in motor, cognitive, and perceptive abilities with age, as well as unfamiliarity with new technologies and motivational barriers) may also have contributed to some of the interventions having been discontinued (Preschl et al., 2011; Wildenbos et al., 2018).

In contrast to the pharmaceutical industry, there is no well-established mechanism for acquiring funding to market and implement eHealth interventions in practice. Hence, of the two interventions that were still available, both had received long-term, external aid from a funding body. Furthermore, the three additional interventions that were exclusively accessible through research also relied on long-term funding. Of course, having aid from national funding bodies was not a guarantee of enduring use - there were also interventions funded by these same types of funding bodies, which were no longer available. Indeed, most evidence-based interventions are funded by short-term, finite grants, centered around the creation of ever more new interventions, leading to the replication and eventual abandonment of increasingly similar interventions. In general, funding bodies focus on new development, rather than sustainability and long-term implementation, meaning most academically-developed interventions reach very few caregivers (Gitlin et al., 2015). Interestingly, only one of the survey respondents mentioned this support from community and government organizations as a facilitator for long-term implementation, though lack of funding in general was mentioned as a barrier. In the theme ‘Iterative Development Process’, a number of the surveyed authors pointed to the creation of a business model early on as important facilitator to implementation. However, the evidence suggests that none of the interventions from the Boots et al. (2014) review developed a self-sustaining, commercial business model, and were instead reliant on external funding. Conversely, there are many commercially-developed interventions on the market that are not scientifically tested for effectiveness (Eysenbach et al., 2002). This is in part caused by the golden standard of randomized controlled trials (RCTs) for eHealth research. While RCTs do provide valuable insights to eHealth effectiveness and mechanisms, they are time-consuming, resource-intensive, and often lacking important, qualitative implementation data (Vernooij-Dassen and Moniz-Cook, 2014). Much like this study's survey respondents, Baker et al. (2014) suggest considering alternative, more efficient research designs. Moreover, there are differing concepts of success for academically versus commercially-developed eHealth interventions: For researchers something is successful if it works, whereas for commercial parties something is successful if it sells. Another potential solution is for policy makers and funding bodies to dedicate more funding to the sustainability and long-term development of evidence-based eHealth interventions. Good examples of recent projects addressing these issues by focusing on improving accessibility of existing eHealth interventions through national implementation platforms include Sweden's Health Innovation Platform (Brown, 2016), Spain's AppSalut (European Innovation Partnership on Active and Healthy Ageing, 2017), and the UK's NHS and NICE collaboration (NHS England, 2018). In this regard, it is also important to note that so far this article has discussed ‘available interventions’, rather than ‘implemented interventions’. While availability can be assessed with an internet search, for an intervention to be called an ‘implemented intervention’, the intervention should be not only proven-effective and available, but also show a good fit with a specific context on the basis of experiential findings concerning what might succeed in that context (Community Tool Box, 2018). Thus, this good fit with clinical practice could also mean funding in routine care instead of funding from a research context.

Articles with lower levels of implementation readiness did not show evidence of enduring use, while the relationship between high levels of reported implementation readiness and enduring use was less clear. The average reported implementation readiness rose from 36% to 52% in the five studies identified by the follow-up search, compared to the original included studies. While it is encouraging that the follow-up average implementation readiness is above 50%, it must also be noted that no follow-up study could be identified for the majority of studies. This is in line with earlier research, indicating that there is a lack of implementation research for eHealth interventions for caregivers of people with dementia (Christie et al., 2018). It would also seem that follow-up research might contribute to lasting use, as the two interventions that were still available were both discussed in follow-up studies, signifying another argument for funding more implementation research. In the theme ‘Integrated Delivery of the Intervention’, survey respondents also cited professionals' lack of training in the eHealth interventions and difficult collaboration with the clinicians and financial gatekeepers (healthcare organizations, health insurers, advocacy groups, as well as business and commercialization partners) as important barriers to successful implementation. eHealth interventions circumvent the normal delivery methods and care structures, leaving many professionals, healthcare organizations, and governing bodies unprepared to adopt and assimilate the interventions and unable to adapt existing structures and norms to incorporate the interventions (Stroetmann, 2013). A recent systematic review has pointed at workload concerns (resulting from both technical problems and the time needed to convert clinical data into digital formats and learn new forms of communication), a lack of incentives, perceived threats to autonomy, liability concerns and lack of organizational support and cooperation as barriers contributing to professionals reluctance to embrace eHealth (Lluch, 2011). Studies have suggested embedding eHealth care skills within training and education for health care professionals (Barakat et al., 2013; van Gemert-Pijnen et al., 2011). Currently, this is difficult in the limited time frame of effectiveness studies and the prevalent ‘design-build-run and see what happens’ approach (van Gemert-Pijnen et al., 2011). Again, funding research into longer-term implementation studies and shedding light on organizational and contextual factors will also help address these professional reluctance issues and aid interventions in sustainably finding their way into practice.

### 4.1 Limitations

The limited participation rate remains low due to the sample constrained by the number of authors included in the Boots et al. (2014) review. This modest participation rate may have several causes. First, researchers may have declined to participate in the study if the technologies used in their interventions were out-of-date, or if their interventions were not implemented. Because of these issues, there is some risk of non-response bias in this study. Additionally, despite contacting multiple authors per interventions, this study only contains information from a single, self-report point of view on the intervention, which could introduce further bias. Furthermore, although a recent review was selected, several of the included publications were quite old. Due to the nature of eHealth is to be expected that some of the older technologies have become outdated and are therefore no longer in use. Next, the ImpRess checklist was not developed to assess the implementation of eHealth interventions, but rather of manualized interventions (Streater et al., 2016). As a result, not all items are optimally suited for assessing eHealth. For instance, it is possible that some of the included interventions did not use manuals, meaning they could potentially not meet the one item on the checklist referring to the manuals. Furthermore, is also important to note that the checklist score is based on what was reported in the article. It is possible the assessed interventions were more implementation-ready than was reported in the article. Moreover, the ImpRess checklist is a newly developed, experimental tool and for this reason has yet not been tested for internal and external validity. Finally, it must be acknowledged that there is a possibility for bias influencing the results, as the authors of this study filled in the ImpRess checklist, and not the authors of the original studies. This was done because it would not be possible to acquire this perspective on all of the included interventions, due to the fact that half of the authors did not reply to the survey request. Nonetheless, the choice was made to use the ImpRess checklist due to its high face validity, its strong base in and synthesis of the existing literature, and the fact that it was viewed as the most suitable tool for taking the varied aspects of implementation readiness into account.

## 5 Conclusions

The available evidence suggested that most interventions in our sample could not be considered ‘implementation-ready’, based on the implementation reporting in the included articles and most eHealth interventions for people with dementia seemed to be unavailable to the caregivers after the effectiveness study. Results from the online search, implementation readiness assessment, and survey could suggest that the presence of long-term funding and considering real-world implementation from the start were the two most important factors determining whether the interventions in this sample were still available. However, the evidence is thin because of this study's missing information and small sample size. Nevertheless, policy makers and funding bodies should consider shifting focus from developing ever more and newer interventions, to funding sustainable implementation of evidence-based interventions, that explore organizational and contextual success factors, from conception to daily practice.

## Funding

This research was carried out as part of the H2020 Marie Skłodowska-Curie Actions Innovative Training Network (ITN) action, H2020-MSCA-ITN-2015, under grant agreement number 676265.

## Declaration of Competing Interest

None.

## Figures and Tables

**Table:** 

Author(s)/year	Description (from Boots et al., 2014)	Citations	Funding	Availability	Follow-up	Survey?
Beauchamp et al., 2005	Caregivers' friend: dealing with dementia: web-based multimedia intervention, text material, and videos. Tailored to the individual through a questionnaire. Three modules; aimed at knowledge, cognitive, and behavioral skills, affective learning. Only online modules and reminder e-mails	189	National Institute of Aging	Not available online	n/a	Yes
Brennan et al., 1995	ComputerLink: website (information, decision support, and communication); enhancing self-care; understanding of AD; promoting health management of the care recipient.	322	National Institute of Aging	Not available online	Payton et al. (1995)	No
Chiu et al., 2009	Caregiver-therapist e-mail support and bilingual information web portal	51	Unknown	Not available online	Chiu and Eysenbach (2011)	No
Coulehan, 2011	Educational and peer- support website.	5	National Institute on Aging (Grant #R43AG026227)	Freely available online	n/a	Yes
Ducharme et al., 2011	Online Stress Management Training Program: individual work + exchange with other caregivers online; seven psycho-educational sessions (60–90 min, once a week); exercises with specific individual objectives and learning activities; online coaches, three times per week online Q&A at set times.	30	Fondation de l'Institut universitaire de gériatrie de Montréal, Desjardins Sécurité Financière and the Social Sciences and Humanities Research Council of Canada.	Available through participating in trial	n/a	Yes
Glueckauf et al., 2004	Support Online (AlzOnline): telephone and Internet: six 45-min live, interactive classes on stress, interpersonal communication + family relationships, emotional well-being and setting/implementing personal caregiving goals; communication via chat box and telephone.	107	State of Florida Department of Elder Affairs and the Robert Wood Johnson Foundation to Robert L. Glueckauf	Freely available online	n/a	No
Kelly, 2003	Link2Care: information library: fact sheets, articles, website links, “Ask the Expert,” personal consultation with technical experts in caregiving, law, and health; peer-moderated support group; personal e-journal; current caregiver news; periodic e-mail communication.	10	Unknown	Not available online	n/a	No
Lai et al., 2013	Gingko: website; 7-week online training workshop + online support forum.	14	Central Research Grant, The Hong Kong Polytechnic University. Grant Number: G-T901	Not available	n/a	No
Lewis et al., 2010	Internet-Based Savvy Caregiver (IBSC) program: Internet-based program: four modules: effects of dementia, taking charge and letting go, providing practical help and managing daily care and difficult behavior; videos, written descriptions, examples of typical caregiver responses + strategies for caregivers to use.	85	NIH	Available through participating in trial	Griffiths et al. (2015)	Yes
Marziali and Garcia, 2011	Online Dementia Caregiver Information Handbook; e-mail list of group members, chat forum, video conferencing link for group meetings, library of educational videos; two groups: (1) CG: access to information handbook and six videos (24/7); (2) VG: psychotherapeutic support group intervention (1 h once a week).	95	Ontario Ministry of Health and Long-Term Care	Available to select hospital staff involved in the research	Nalder et al. (2018)	Yes
Torp et al., 2008	ACTION: ICT course, three 3-h classes (in 3-week period): disease information, care for patients, nutrition, social services, being a family carer + online discussion forum; option to interact with other participants via videophone + 3-h Internet training in using and collecting information	41	Directorate for Health and Social Affairs and the Norwegian Association of Local and Regional Authorities	Not available online	n/a	No
van der Roest et al., 2010	DEM-DISC (Dementia-specific Digital Interactive Social Chart): clarifying needs to specific demands in a three-step procedure, general and tailored information on available dementia care and welfare services, information on caregiving.	30	Dutch Ministry of Economic Affairs under contract BSIK 03025, Dioraphte Foundation, RCOAK, NHDI, Foundation Het Zonnehuis, Province Noord-Holland and Stichting Alzheimer & Neuropsychiatrie Foundation	Not available online	Van Mierlo et al. (2015)	Yes

Note: Adapted from Boots et al. (2014).
